# Editorial: Novel research on metabolites secreted by gram-positive bacteria

**DOI:** 10.3389/fmicb.2023.1237790

**Published:** 2023-06-27

**Authors:** José E. Barboza-Corona, Dennis K. Bideshi, Hyun-Woo Park, Rubén Salcedo-Hernández

**Affiliations:** ^1^Departamento de Alimentos, Posgrado en Biociencias, Universidad de Guanajuato, Campus Irapuato-Salamanca, Irapuato, Guanajuato, Mexico; ^2^Department of Biological Sciences, Program in Biomedical Sciences, California Baptist University, Riverside, CA, United States

**Keywords:** bacteria, secretion proteins, antimicrobial peptides, hydrolytic enzymes, secondary metabolites

Bacteria play essential roles in nature and have a ubiquitous distribution. Their mutualistic, commensal, and parasitic associations with humans, animals, fungi, and plants have been extensively documented. Many of these synergistic interactions are mediated by biomolecules they secrete, which are of industrial and pharmaceutical interests. Bacteria are classified as Gram-positive or Gram-negative based primarily on their cell wall structure. Gram-positive bacteria secrete metabolites directly into culture media, facilitating rapid recovery for downstream applications. The papers presented in “*Novel research on metabolites secreted by gram-positive bacteria*” include original research, reviews, and *in silico* analyses of compounds with antimicrobial and bioactive properties produced by Gram-positive bacteria from different ecological niches.

In the first paper, *In silico Prediction and Exploration of Potential Bacteriocin Gene Clusters Within the Bacterial Genus Geobacillus*, Egan et al. utilized the BAGEL3 software to scan publicly available genomes that harbor putative bacteriocin gene clusters. They identified the endospore-forming genus *Geobacillus* as a potential source of novel bacteriocins that could have commercial and therapeutic value in treating antibiotic-resistant bacteria of clinical significance.

Recognized as probiotic bacteria, the ability of *Lactococcus* and *Lactobacillus* species to support immune balance and gut epithelial integrity is of crucial interest. Sokovic Bajic et al. showed that *Lactobacillus brevis* BGZLS10-17, a strain isolated from Zlatar cheese, can produce high levels of gamma amino butyric acid (GABA) and reduced adhesion of *Salmonella enterica* and *Escherichia coli* to colonocyte-like Caco-2 cells. Additionally, the monolayer of differentiated Caco-2 cells treated with GABA-containing supernatants alleviated an inflammatory response and increased the production of tight junction proteins. Their findings demonstrated the possibility of developing novel functional dairy products and beverages that can be useful in modulating and suppressing immunological responses that give rise to clinically significant inflammatory conditions in the digestive system.

One of the technical problems associated with acquiring sufficient bacteriocins for basic and applied studies is that they are synthesized in low amounts by the native producer strains. Within the *Lactococcus* group, Telke et al. reported that *Lactococcus garvieae* KS1546 produces a bacteriocin called Garvicin, that is active against clinically significant bacteria. By combining different strategies, including genetic engineering, and modifying culture media and conditions, they produced a KS1546 strain that exhibited a 2000-fold increase in Garvicin synthesis.

Optimizing the growth of beneficial microbes in natural niches using “helper” microbes is also of significant interest. Toward this end, Xu et al. showed that adding *Lactobacillus plantarum* and *L. buchneri* to corn silage favored the proliferation of native beneficial *Lactobacillus* species while suppressing the growth of other species. Interestingly, the study also showed that corn silage treated with *L. plantarum* and *L. buchneri* increased metabolite yields of antimicrobial and antioxidant activities and bioactive compounds that can reduce cholesterol and help control depression.

Another intriguing microbial group is represented by *Bacillus* spp., which produces metabolites of interest to the food, pharmaceutical, and veterinary industries. Taking this into account, Caulier et al. provide a timely review of antimicrobial metabolites produced by the *Bacillus subtilis* group and propose a classification system based on the biosynthetic pathways, i.e., ribosomal peptides (RPs), non-ribosomal peptides (NRPs), volatiles, polyketides (PKs), compounds, and hybrids between PKs and NRPs, and the chemical nature of these microbes.

In addition to antimicrobials, *Bacillus* and actinobacteria species synthesize compounds beneficial to plants, and their symbiotic interactions with plants result in the generation of biomolecules with broad applications. In their paper titled, *Bioactive products from plant-endophytic gram-positive bacteria*, Ek-Ramos et al. provide a comprehensive review of metabolites produced by *Bacillus* and actinobacteria endophytes. These metabolites include aromatic compounds, lipopeptides, plant hormones involved in promoting plant growth, polysaccharides, enzymes, and other compounds that potentially have applied use in agriculture and human and veterinary medicine.

Compared to hydrated biomes, relatively fewer studies have been conducted on microbial competition in arid soils. The study by Nasfi et al. found that the dominant species in arid rhizosphere soil were *Bacillus* sp. Interestingly, 93% of the isolates were active against Gram-positive and Gram-negative bacteria. Among these isolates was *Bacillus* sp. M2 that showed promising antimicrobial activities against Gram-negative bacteria. The inhibitory activity was attributed to the organic compound 1-acetyl-β-carboline, which also has antitumor, antiviral, and antiparasitic properties. *Bacillus* sp. M2 also produced fungicides such as bacillomycin, fengycin and surfactins, which could be helpful in controlling other organisms. Regarding the latter, Rodriguez et al. showed that *Bacillus atrophaeus* produces lipopeptides, including surfactins, fengycins, and bacillomycins that damage the cuticle membrane and decrease the foraging capability of *Rhopalosiphum padi*, an aphid that elicit significant economic losses to cereal crops.

Gram-positive bacteria can also be used as microbial cell factories to produce metabolic precursors of biomolecules that can treat human diseases such as cancer. Abdallah et al. successfully produced taxadiene, a precursor of taxol, through metabolic engineering of *B. subtilis* 168 by transforming this strain to produce taxadiene synthetase (TXS). TXS converts geranylgeranyl pyrophosphate (GGPP) to taxa-4,11-diene. Moreover, by further engineering the TXS-producing *B. subtilis* to express the *ispA* and *crtE* genes and a synthetic operon that increases the supply and flux of the GGPP precursor, they increased the production of taxadiene 81-fold. Finally, small molecules, such as pipecolic acid or L-PA, have gained importance in the pharmaceutical and chemical industries, as these compounds are important in synthesizing specific amino acids, neurotransmitters, and in plant immune defenses against bacterial pathogens. These metabolites can be synthesized in *Corynebacterium glutamicum*. Understanding L-PA's physiological function in microbial factories is essential to the commercial development of these microbial factories. Pérez-Garcia et al. showed that the external addition of L-AP to a culture medium or its *de novo* synthesis is advantageous for bacterial growth under hyper-osmotic stress conditions and that proline permease ProP and the mechanosensitive channel YggB are involved in the export and import of L-PA.

## Conclusion

In conclusion, Gram-positive bacteria continue to be a resource for secreted biomolecules, including antimicrobial metabolites, lipopeptides, metabolites (GABA, 1-acetyl-β-carboline), and organic acids, which not only naturally influence the establishment and maintenance of diverse ecological niches, but also for multiple applications in human and animal health, plant crop protection, and the food industry. Current developments in genetic manipulations focused on engineering Gram-positive bacteria to be used as microbial factories to express proteins or metabolites with biotechnological applications are intriguing. Indeed, synthetic biology will prove to be indispensable in translational endeavors. Although this Research Topic highlighted, arguably, of the most significant resource species, i.e., *Lactococcus, Lactobacillus*, and *Bacillus*, it is clear that current and future exploration of less studied microbes, including *Geobacillus*, will undoubtedly identify novel metabolites secreted by a broad range of Gram-positive bacteria that can be exploited for applied purposes.

## Author contributions

JB-C and DB wrote the editorial. All authors contributed to the article and approved the submitted version.

